# How to Properly Diagnose and Treat Immune-Related Adverse Events During Immunotherapy in Patients with Cancer: Discussion Between Specialists

**DOI:** 10.3390/cancers18030523

**Published:** 2026-02-05

**Authors:** Angioletta Lasagna, Paolo Sacchi

**Affiliations:** 1 Medical Oncology Unit, Fondazione IRCCS Policlinico San Matteo, 27100 Pavia, Italy; 2 Division of Infectious Diseases I, Fondazione IRCCS Policlinico San Matteo, 27100 Pavia, Italy; p.sacchi@smatteo.pv.it

## 1. Introduction

Immune checkpoint inhibitors and other immune-based cancer therapies have profoundly transformed the treatment of solid and hematological malignancies, leading to durable responses and significant survival benefits in a growing number of patients. Alongside these achievements, however, the activation of the immune system has introduced a unique spectrum of toxicities, known as immune-related adverse events (irAEs), which represent a major challenge in contemporary oncology practice [1,2,3]. IrAEs differ substantially from toxicities associated with conventional anticancer treatments. They may involve virtually any organ system, present with heterogeneous and sometimes atypical clinical manifestations, and occur at unpredictable time points during or after therapy [4,5,6,7]. As immunotherapy continues to expand into earlier disease stages, combination strategies, and patient populations with pre-existing comorbidities, the incidence and clinical complexity of irAEs are expected to increase further. Consequently, the ability to promptly recognize, accurately diagnose, and effectively manage immune-mediated toxicities has become an essential component of cancer care.

This Special Issue, “How to Properly Diagnose and Treat Immune-Related Adverse Events during Immunotherapy in Patients with Cancer: Discussion between Specialists”, brings together original research articles and reviews that reflect the current multidisciplinary understanding of irAEs. The collected works address clinical presentation, diagnostic approaches, management strategies, predictive factors, and patient-centered considerations, offering a broad and integrated overview of this rapidly evolving field.

## 2. Key Themes

A central theme emerging from this Special Issue is the importance of real-world evidence in characterizing immune-related toxicities. Data derived from routine clinical practice complement findings from clinical trials by capturing a broader patient population and reflecting everyday diagnostic and therapeutic challenges [1,6]. These observations emphasize that the incidence, severity, and clinical course of irAEs may vary according to tumor type, treatment regimen, and individual patient characteristics [2,4].

Another key aspect concerns organ-specific immune-mediated toxicities, which remain among the most challenging complications of immunotherapy. Endocrine, neurological, hepatic, and gastrointestinal manifestations are particularly relevant due to their potential severity and diagnostic complexity [3,5,7]. These toxicities often mimic other conditions, such as infection or disease progression, underscoring the need for a high index of suspicion and early multidisciplinary involvement.

The role of imaging and advanced diagnostic techniques is also highlighted. Radiological and nuclear medicine tools are increasingly used to detect immune-mediated inflammation and to support differential diagnosis [4,5]. At the same time, their limitations—especially the difficulty in distinguishing inflammatory changes from tumor progression—stress the necessity of integrating imaging findings with clinical and laboratory data.

From a therapeutic perspective, the management of irAEs continues to evolve. While corticosteroids remain the cornerstone of treatment for moderate to severe toxicities, there is growing interest in steroid-sparing and targeted immunosuppressive strategies aimed at reducing long-term complications and preserving antitumor immune activity [1,2]. The balance between effective toxicity control and maintenance of treatment efficacy represents a critical and ongoing challenge.

The identification of predictive and prognostic factors for irAEs is another emerging area of research. Exploratory studies on clinical parameters, systemic inflammatory markers, and immune-related biomarkers suggest the possibility of earlier risk stratification and more proactive management [6], although further validation is required before widespread clinical implementation.

Finally, this Special Issue underscores the importance of patient-centered care, particularly in complex clinical scenarios such as patients with pre-existing autoimmune diseases [3,6]. Patient education, shared decision-making, and clear communication regarding risks and benefits are essential elements of safe and effective immunotherapy.

## 3. Conclusions

The contributions included in this Special Issue collectively demonstrate that irAEs are an integral aspect of modern cancer immunotherapy. Their heterogeneous nature and potential severity require a coordinated, multidisciplinary approach that involves oncologists, organ-specific specialists, radiologists, and other healthcare professionals. Despite substantial progress, important gaps in knowledge remain, including the lack of standardized diagnostic criteria, validated predictive biomarkers, and consensus-driven management algorithms for many irAEs. Addressing these unmet needs will be essential as immunotherapy continues to expand and patient survival improves. By providing an integrated overview of current challenges and emerging strategies, this Special Issue aims to support clinicians and researchers in improving the diagnosis, management, and prevention of immune-related adverse events, ultimately contributing to safer and more sustainable use of cancer immunotherapy.

## Data Availability

Not applicable.

## Abbreviations

The following abbreviations are used in this manuscript:

irAEs	immune-related adverse events

## References

[1] Coniac S., Costache-Outas M.C., Antone-Iordache I.L., Anghel A.V., Bobolocu M.A., Zamfir A., Liscu H.D., Ionescu A.I., Badiu C. (2025). Real-World Evaluation of Immune-Related Endocrinopathies in Metastatic NSCLC Patients Treated with ICIs in Romania. Cancers.

[2] Syed S., Hines J., Baccile R., Rouhani S., Reid P. (2024). Studying Outcomes after Steroid-Sparing Immunosuppressive Agent vs. Steroid-Only Treatment for Immune-Related Adverse Events in NSCLC and Melanoma. Cancers.

[3] Lopez-Olivo M.A., Kachira J.J., Buni M., Kim S.T., Lu H., Tayar J.H., Duhon G.F., Ruiz J.I., Bingham C.O., Calabrese C. (2023). Learning Needs of Patients with Cancer and a Pre-Existing Autoimmune Disease Who Are Candidates for Immune Checkpoint Inhibitors. Cancers.

[4] Pozzessere C., Mazini B., Omoumi P., Jreige M., Noirez L., Digklia A., Fasquelle F., Sempoux C., Dromain C. (2024). Immune-Related Adverse Events Induced by Immune Checkpoint Inhibitors and CAR-T Cell Therapy: A Comprehensive Imaging-Based Review. Cancers.

[5] Karlsen W., Akily L., Mierzejewska M., Teodorczyk J., Bandura A., Zaucha R., Cytawa W. (2024). Is 18F-FDG-PET/CT an Optimal Imaging Modality for Detecting Immune-Related Adverse Events after Immune-Checkpoint Inhibitor Therapy? Pros and Cons. Cancers.

[6] Nelli F., Fabbri A., Virtuoso A., Giannarelli D., Giron Berrios J.R., Marrucci E., Fiore C., Ruggeri E.M. (2024). Early Changes in LIPI Score Predict Immune-Related Adverse Events in Advanced NSCLC. Cancers.

[7] Lasagna A., Sacchi P. (2024). The ABC of Immune-Mediated Hepatitis during Immunotherapy in Patients with Cancer: From Pathogenesis to Multidisciplinary Management. Cancers.

